# Validation of ICD-10 codes used to identify the main reason for hospitalization due to hyponatremia in Sweden

**DOI:** 10.1371/journal.pone.0318927

**Published:** 2025-02-14

**Authors:** Cecilia Bergh Fahlén, Issa Issa, Fredrik Sahlander, Jonatan D. Lindh, Jan Calissendorff, Henrik Falhammar, Jakob Skov, Buster Mannheimer

**Affiliations:** 1 Department of Clinical Science and Education at Södersjukhuset, Karolinska Institutet, Stockholm, Sweden; 2 Department of Internal Medicine, Section of Diabetes and Endocrinology, Södersjukhuset, Stockholm, Sweden; 3 Department of Molecular Medicine and Surgery, Karolinska Institutet, Stockholm, Sweden; 4 Department of Medicine, Falu Hospital, Falun, Sweden; 5 Center for Clinical Research Dalarna, Uppsala University, Falun, Sweden; 6 Division of Clinical Pharmacology, Department of Laboratory Medicine, Karolinska University Hospital Huddinge, Karolinska Institutet, Stockholm, Sweden; 7 Department of Endocrinology, Metabolism and Diabetes, Karolinska University Hospital, Stockholm, Sweden; 8 Department of Medicine, Karlstad Central Hospital, Karlstad, Sweden; Bursa Ali Osman Sonmez Oncology Hospital, TÜRKIYE

## Abstract

**Purpose:**

Hyponatremia is the most common electrolyte disorder, with potentially serious consequences. Studies validating hyponatremia used as a principal diagnosis are lacking. Studying principal diagnoses is important since it indicates the main reason for inpatient care. The aim of this study was to assess the validity of principal diagnoses of hyponatremia according to the International Classification of Diseases, tenth revision (ICD-10) in patients discharged from hospital.

**Methods:**

Three independent reviewers retrospectively analyzed medical records of 428 hospitalized patients, ≥18 years old, discharged with a principal diagnosis of either hyponatremia or syndrome of inappropriate anti-diuretic hormone secretion (SIADH) from three different hospitals during 2014–2017. The positive predictive value of hyponatremia as a principal diagnosis was calculated.

**Results:**

The median age of the studied population was 73 (IQR 64–81) years and 69% were women. The range of sodium levels was < 100 to 139 mmol/L. The mean sodium level was 119 mmol/L. Of the patients 27% were treated in an intensive care unit (ICU)/high dependency unit (HDU). Hyponatremia was deemed to be the principal cause of inpatient care in 411 out of 428 patients, corresponding to a positive predictive value for hyponatremia as a principal diagnosis of 96% (CI 95% 94–98).

**Conclusions:**

The current study showed that 19 diagnoses out of 20 were correct implying a high validity. Thus, a principal diagnosis of hyponatremia, appears to be a useful measure in clinical and epidemiological studies aiming to target a clinically relevant consequence of hyponatremia in hospitalized patients.

## Introduction

Hyponatremia is the most common electrolyte abnormality encountered in health care [1]. It is seen in a wide variety of chronic medical conditions, such as heart, renal, and liver failure, as well as in acute conditions, such as diarrhea and vomiting [2]. Hyponatremia can also be iatrogenic, caused by the intake of common drugs, e.g., thiazide diuretics and antidepressants [3–5]. Severe complications of hyponatremia include coma, permanent brain damage, respiratory arrest, brain stem herniation, and death [6,7]. Studies show that at least 30% of patients in medical, surgical, and psychiatric wards are hyponatremic [8]. One third of all hyponatremia cases result from syndrome of inappropriate anti-diuretic hormone secretion (SIADH) [9]. Advanced age, female sex and low body mass are important risk factors for developing hyponatremia [7,10].

Validity refers to how well a measure assesses what it is intended to measure [11]. Consequently, an evaluation of diagnosis codes in medical records is primarily conducted as quality control. The purpose here is to determine if the principal diagnosis is accurately chosen and reliable [12,13].

Hospitalization is a clinically significant event, often indicating severe disease and it also entails a large cost for society. The principal diagnosis, reflecting the main reason for hospitalization, may therefore be a clinically relevant outcome for register-based studies.

Principal diagnoses have in fact previously been used to investigate the association between drug groups and hospitalization due to hyponatremia [4,14–21], investigating mortality after being hospitalized due to hyponatremia [7] and to which extent clinicians discontinue thiazides after being hospitalized due to hyponatremia [5]. However, these studies rely on the quality of the principal diagnoses: in essence, their validity.

The aim of this study was to assess the validity of ICD-10 (International Classification of Diseases) codes for hyponatremia as principal diagnoses in patients discharged after hospitalization.

## Materials and methods

### Study design and population

This study is a Swedish population based retrospective validation study of hyponatremia as principal diagnosis at discharge. We included 428 patients discharged from somatic care wards in three different hospitals Södersjukhuset (a metropolitan teaching hospital, n = 200) in Stockholm, Karlstad Hospital (a regional hospital, n = 109) and Falu Hospital (a regional hospital, n = 119). We also included patients who had received intensive care. The catchment area for Södersjukhuset was approximately 750 000 inhabitants, Falu Hospital nearly 200 000 inhabitants and Karlstad Hospital services roughly 275 000 inhabitants. We enrolled adult patients, i.e., 18 years or older, with an ICD-10 principal diagnosis of hyponatremia (E87.1/E87.1B) or SIADH (E22.2) between 2014 and 2017. All the adult patients are included from Södersjukhuset during the years 2015–2016, from Karlstad Hospital 2014–2015 and Falu Hospital 2014–2017.

The data were accessed between January 2019 and December 2022.

### Validation of hyponatremia as a main diagnosis

By using the Swedish personal identity number, three independent reviewers (CBF, II, BM) analyzed the inpatient medical records of 428 individuals discharged with a principal diagnosis of hyponatremia during 2014–2017. More specifically, CBF and II reviewed all medical records. In cases where the conclusions were not aligned, the third reviewer (BM) reviewed the medical records and finally decided on whether to confirm or reject the original diagnosis. The retrieved records included enrollment note, daily notes, discharge note, drug list from the enrollment and laboratory lists, generated as part of routine care.

The present study was based on the ICD-10 codes for principal diagnosis. According with instructions from the Swedish Board of Health and Welfare, aiming to clarify the guidelines from WHO’s International Classification of Diseases and Related Health Problems, Tenth Revision (ICD-10), the principal diagnosis is established at the end of the (inpatient) care contact. To evaluate the principal diagnosis, these guidelines were followed. Thus, a diagnosis was considered a principal diagnosis if it was recognized as the main reason for a healthcare contact, established by the time of discharge. For this reason, the diagnosis that appears to be the reason for hospitalization is usually chosen as the principal diagnosis. A condition that occurs or is detected during the care contact should be selected as the principal diagnosis if it consumes more resources than the condition that is the initial cause of the care contact. If there is more than one reason for the care contact, the condition that used most resources should be selected as the principal diagnosis. A complication caused by the healthcare contact or treatment can never be the principal diagnosis regardless of resources consumed [12].

A principal diagnosis of hyponatremia was considered valid if it was the main reason of the inpatient care as described above.

Sodium and glucose concentrations were noted, both the first and lowest sodium concentrations, as well as related glucose concentrations measured within 1 hour. Sodium concentrations were corrected for glucose concentrations using a correction factor of 2.4 mmol/L increase in sodium concentration per 100 mg/dL as suggested by Hillier et al. [22]. In cases where glucose measurement was not available within 1 hour, the sodium concentration was based on the sodium concentration alone if evidence for uncontrolled diabetes (obvious hyperglycemic symptoms/ketoacidosis and/or HbA1c > 80 mmol/mol and/or glucose concentrations > 15 mmol/L during the hospitalization period) was lacking. The sodium and glucose values were analyzed either in plasma or arterial blood samples. Both arterial and venous blood gas samples were accepted.

We calculated the positive predictive value (PPV) for patients with a principal discharge diagnosis of hyponatremia/SIADH. PPV was calculated as the ratio of discharges with a correct principal diagnosis of hyponatremia/SIADH confirmed through the authors’ review versus all discharges with a principal diagnosis of hyponatremia/SIADH. If any of the criteria above was not fulfilled the discharge diagnosis was classified as incorrect. The PPV is then presented in percentage with a 95% CI.

### Statistical analysis and variables

We used descriptive statistics such as mean, median, ranges and standard deviations as appropriate. PPV of patients with a principal discharge diagnosis of hyponatremia/SIADH was determined. To perform the analyses, we used the statistical programs Excel Microsoft 365 version 2310 and R version 4.3.1.

### Ethical approval

The study was approved by the Regional Ethical Review Board in Stockholm. The authors that collected the data had access to the identity of the participants in their local hospital during data collection.

Informed consent was waived by the Ethical Committee due to the retrospective nature of the study.

## Results

We included in total 428 patients from Södersjukhuset (n = 200), Karlstad Hospital (n = 109) and Falu Hospital (n = 119).

The range of sodium concentrations at admission ranged from < 100 to 139 mmol/L (the patient with sodium of 139 mmol/L, had lower levels when were treated earlier at another hospital), and the lowest levels during hospitalization between < 100 and 135 mmol/L, respectively. The mean (SD) sodium concentration (first) was 120 (±6) mmol/L and the lowest 115 (±8) mmol/L. The mean (SD) of glucose concentrations used to correct the sodium values was 7 (±3) mmol/L. Patients with severe (sodium < 125 mmol/L) and very severe (sodium < 115 mmol/L) hyponatremia, were 54% (231/425) and 31% (130/425) of the patients, respectively.

The median age was 73 (IQR 64–81) and, 69% were women. The mean caretime at the hospital was 5 days. Of the patients 27% were treated in ICU/HDU due to the severe hyponatremia concentrations and/or related comorbidities. The mean (SD) sodium concentration at admission if hospitalized in ICU/HDU was 113 (±6) mmol/L. The most frequent comorbidity was hypertension (n = 284) followed by chronic obstructive pulmonary disease/asthma, diabetes, malignant disease, previous history of a cerebrovascular incident, ischemic heart disease and heart failure (Table 1). Each patient consumed regularly on average 7 prescribed drugs at admission.

**Table 1:** Patient characteristics among the study population (n = 428).

Variables	
Median age (IQR)	73 (64–81)
Number (%) of women	295 (69)
Mean (SD) first sodium at admission to hospital	120 (±6) mmol/L
Mean (SD) lowest sodium during hospitalization	115 (±8) mmol/L
Number (%) of individuals treated in intensive care	118 (27)
*Diagnosis codes (ICD-codes)*	*n (%)*
Hyponatremia (E871B)	368 (86)
Hypoosmolality and hyponatremia (E871)	38 (8.9)
SIADH* (E222)	22 (5.1)
*Comorbidities*	*n (%)*
Hypertension	284 (66)
Chronic obstructive pulmonary disease/asthma	81 (19)
Diabetes	79 (18)
Previous history or present malignant disease	71 (17)
Previous history of cerebrovascular incident	65 (15)
Ischemic heart disease	63 (15)
Heart failure	61 (14)

*Syndrome of inappropriate anti-diuretic hormone secretion.

After reviewing the journals, we found that 411/428 of the principal diagnosis were correct resulting in a positive predictive value of 96% (CI 95% 94–98). The characteristics of the 17 false positive diagnoses are shown in Table 2. According to the current validation, the diagnosis most erroneously misplaced for hyponatremia were renal failure, heart failure and urinary tract infections.

**Table 2:** The final diagnoses of the 17 false positive diagnoses with corresponding sodium values.

Diagnosis	First sodium level	Lowest sodium level
Renal failure	125	–
Renal failure	130	–
Renal failure	133	–
Heart failure	120	–
Heart failure	128	–
Urinary tract infection	122	–
Urinary tract infection	134	132
Anemia	124	–
Chronic obstructive pulmonary disease	126	125
Cortisol insufficiency	126	–
Dehydration	133	122
Edema	132	–
Malignant lung disease	126	124
Neutropenia	131	–
Pneumonia	134	–
Respiratory insufficiency	133	131
Suspected tuberculosis	122	121

## Discussion

This retrospective validation study is the first of its kind, focusing on the evaluation of hyponatremia as a principal diagnosis. The results showed that 96% of patients discharged with hyponatremia as a principal diagnosis received the correct diagnosis.

There is a need for a better understanding of the underlying conditions of hyponatremia. One important step towards this goal is the efficient use of population-based registers, which in turn demands reliable outcomes. Hyponatremia as the principal diagnosis has a large potential in this regard. However, a prerequisite for its use is that it is valid, in essence that it is in fact measuring what it is supposed to measure which is the purpose of the current paper. ICD-codes have previously been used as a proxy for hyponatremia in register-based outcome studies. However, evidence shows that the validity of ICD-codes to subnormal sodium concentrations is modest. Holland-Bill et al included patients discharged with any hyponatremia diagnosis, not only principal diagnosis, and showed that ICD-10 codes for hyponatremia in the Danish National Registry of Patients have high specificity (≥99.8%) but very low sensitivity (1.8%) [23]. A Canadian study was restricted to elderly patients, 66 years or older, and to serum sodium concentrations at the time of emergency department contact or at hospital admission. That study showed that the hyponatremia ICD-10 code differentiates between two patient groups with measurements of serum sodium at the emergency department and at hospital admission. However, these codes underestimated the true incidence of hyponatremia due to low sensitivity (7.5% at presentation to the emergency department and 10.6% at hospital admission). The positive predictive value for the emergency department was 96.4% and 82.3% for hospital admission. Distinctions between principal and secondary diagnosis was not considered [24]. Finally, Movig et al observed that ICD codes for hyponatremia at a single hospital were highly specific but demonstrated low sensitivity. The sensitivity of hyponatremia ICD codes was not higher than around 30% for patients with severe hyponatremia (sodium ≤ 115 mmol/L) and corresponding specificities were very high (>99%) [25].

Although sodium values as well as ICD-codes as a surrogate for sodium concentrations may be useful outcomes in research, they may not always be clinically relevant. Though the clinical significance of secondary diagnosis may vary the main reason for hospitalization is important representing a condition associated with more severe disease that demands the resources associated with inpatient care.

Since the review was limited to patients diagnosed with hyponatremia, we could only estimate the positive predictive value (PPV). A more detailed and complete validation including the remaining measures of validity, i.e., negative predictive value (NPV), sensitivity and specificity, was not feasible because it would have required a review of all discharge summaries discharge summaries at Södersjukhuset, Karlstad- and Falu Hospitals during the full study period. With a total catchment area of 1,275,000 individuals the total number of inpatient medical files in need for review would have amounted to several tens of thousands. Unfortunately, this approach was therefore not feasible. Hence, the current study cannot rule out the possibility of a certain proportion of hospitalizations due to hyponatremia were wrongly diagnosed (false negative assessments). However, at the point of discharge from inpatient care, the physician responsible for the inpatient care is obliged to judge the number one main reason for hospitalization. In contrast, hospitalizations with one or several measurements of sodium below the reference interval in a patient where the main reason for care is derived from a totally different area such as an acute cardiovascular disease or infection may not to a similar extent led to a diagnosis of hyponatremia.

A strength of the current study is that we included three hospitals of different sizes, from different parts of Sweden. The PPV for Södersjukhuset, Karlstad- and Falu Hospital respectively were approximately 96% each, similar to the overall PPV. The participation of additional hospitals would of course increase the generalizability of the results further. To avoid/reduce systematical errors related to interpretation we had three independent reviewers. All with several years of clinical practice within the area of internal medicine which increased the precision of the assessments.

We cannot exclude that the true PPV may deviate from the results in the current study. However, when planning the study, a power analysis was performed. Based on the presumptions on a true proportion of correct diagnosis of 0.9, a sample size of 414 could result in a 95% confidence interval with a total width of 0.06.

## Conclusion

The current study shows that the positive predictive value for hyponatremia used as a principal diagnosis was 96%. Thus, hospitalization due to hyponatremia appears to be a useful measure in epidemiological studies aiming to target a clinically relevant consequence of hyponatremia.

## Supporting information

S1 File Supporting information file. (XLSX)
